# Supplementary material for: Emetine Synergizes with Cisplatin to Enhance Anti-Cancer Efficacy against Lung Cancer Cells
Source: Int J Mol Sci. 2019 Nov 25;20(23):5914. doi: 10.3390/ijms20235914 (PMC6928603; doi:10.3390/ijms20235914)
Supplement: Supplementary file 1 [file ijms-20-05914-s001.pdf]

Supplementary Materials

Supplementary Data

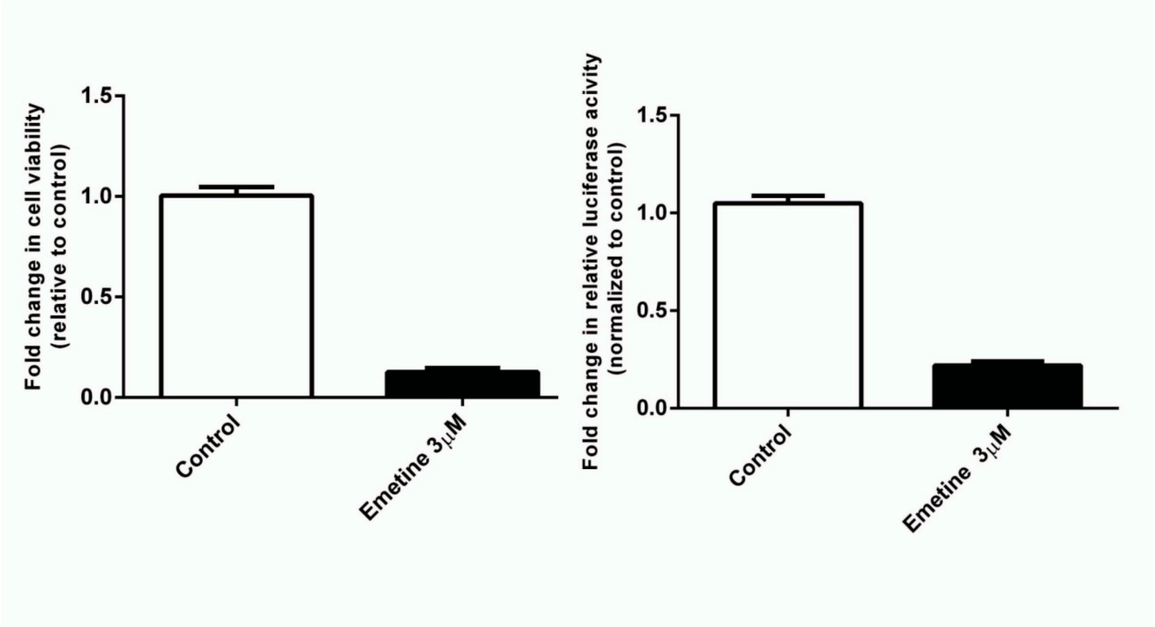

Figure S1. A Wnt-responsive STF luciferase reporter assay was used to identify compounds that inhibited Wnt signaling. We treated Huh6-M50 cells with emetine at a concentration of 3 μM for 72 hours and then measured cell viability by MTS assays (A). Huh6/M50 cells expressing high levels of luciferase activity were established as the cell model for drug screening. Huh6/M50 cells were treated with YC-1 at a concentration of 3 μM for 6 hours, and the inhibitory rate of luciferase activity was then analyzed (B)

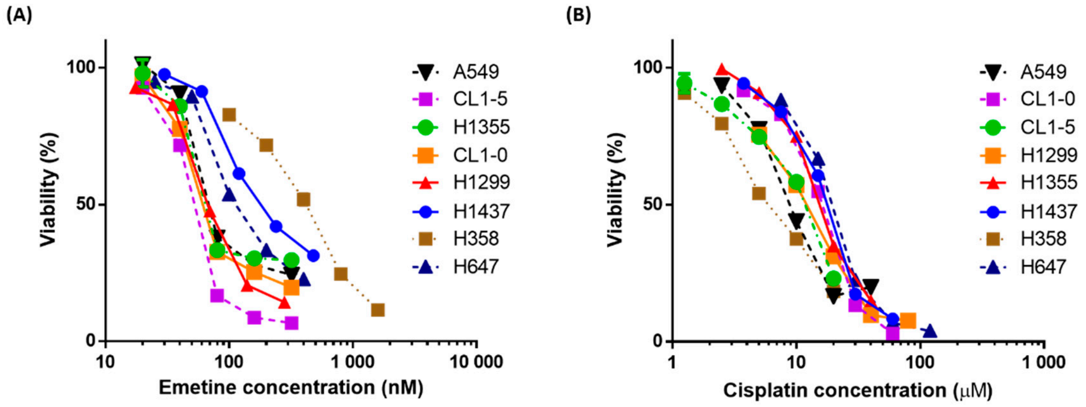

Figure S2. Dose-response curves of lung cancer cell lines treated with cisplatin and emetine. (A) Cells were treated with different concentrations of cisplatin for 48 hours. (B) Cells were treated with different concentrations of emetine for 48 hours. Cell viability was measured using an MTS assay. The data are expressed as the means ± SEMs from three independent experiments performed in triplicate.

Table S1. Estimated 50% inhibitory concentration (IC_{50}) in cultured lung cancer cell lines.

Cultured cell line	Estimated IC_{50} value		
	Emetine (nM)	Cisplatin (μM)	Cisplatin (μM) /Emetine (40 nM)
**A549**	87.88	9.41	5.18
**CL1-0**	72.25	15.34	4.75
**CL1-5**	51.74	10.65	1.84
**H1299**	72.89	11.48	ND
**H1355**	86.94	15.86	6.04
**H1437**	211.1	16.96	9.12
**H358**	384.5	6.40	0.94
**H647**	133.6	19.06	ND

The 50 percent inhibitory concentration (IC_{50}) values were estimated from the log concentration effect curves and nonlinear regression analysis. Combined data from at least three independent experiments are presented. ND, not determined

Table S2. Combination index values obtained at ED50, ED75 and ED90. The mean of the three values is reported in the last row.

	A549	CL1-0	CL1-5	H1299	H1355	H1437	H358	H647	CL1-0/CDDP
**ED50**	0.837	0.639	0.715	1.047	0.863	0.730	0.553	1.084	0.777
**ED75**	0.786	0.590	0.564	1.011	0.718	0.416	0.586	0.801	0.616
**ED90**	0.739	0.579	0.445	0.982	0.606	0.331	0.621	0.594	0.489
**Mean**	**0.788**	**0.602**	**0.575**	**1.013**	**0.729**	**0.492**	**0.587**	**0.827**	**0.627**

Combination index (CI) values were calculated using the Chou-Talalay method. Combined data from at least three independent experiments are presented. ED_{50}, 50% effective dose. ED_{75}, 75% effective dose. ED_{90}, 90% effective dose.

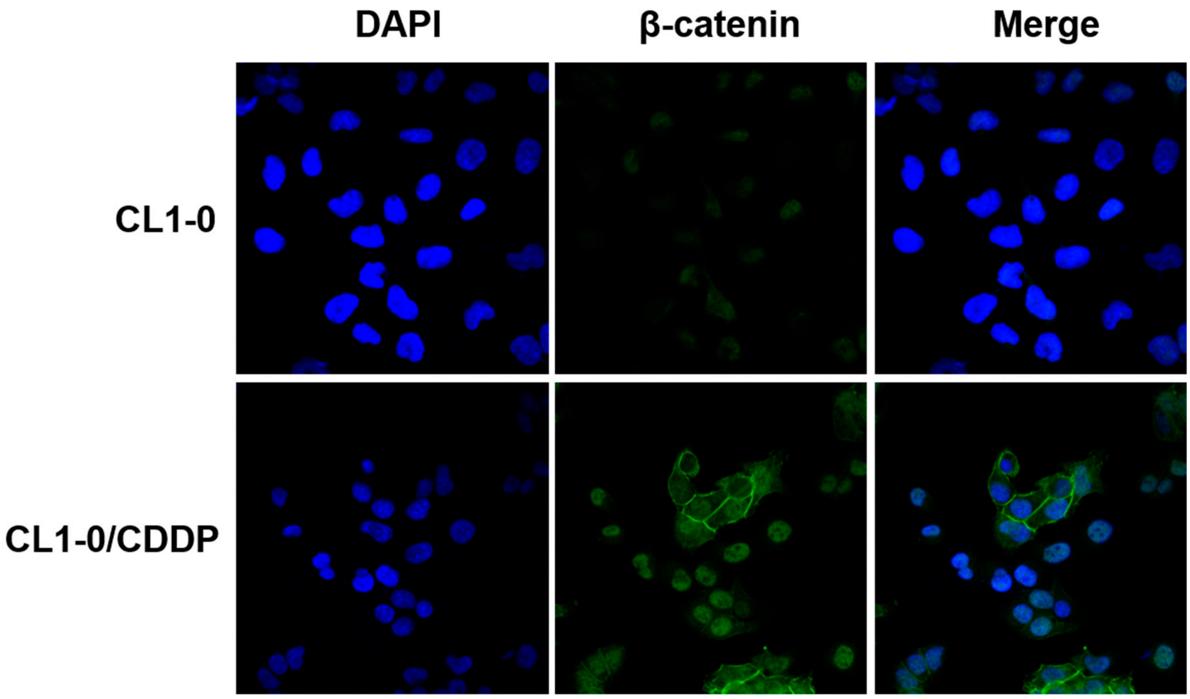

Figure S3. β-Catenin expression is upregulated in the cisplatin-resistant subpopulation of CL1-0 cells. Confocal microscopy analysis of CL1-0 cells and the cisplatin-resistant subpopulation of CL1-0 cells (CL1-0/CDDP). Immunofluorescence staining of β-catenin (green) was performed, and DAPI (blue) was used to counterstain nuclei.

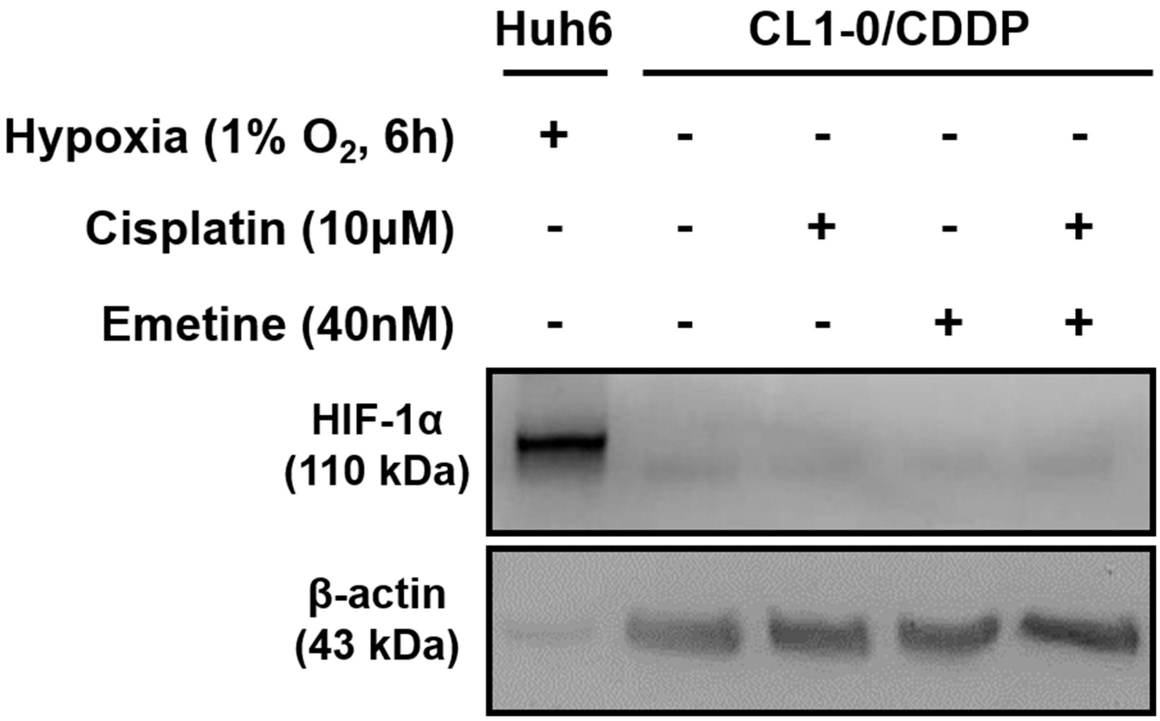

Figure S4. Emetine's antiproliferative effect is HIF-1α-independent. CL1-0 cells in the cisplatin-resistant subpopulation (CL1-0/CDDP) were treated with the indicated concentrations of cisplatin or emetine for 48 hours. The protein expression of HIF-1α was examined by Western blotting. Huh6 cells under hypoxic conditions (1% O_2, 6 hours) served as the positive control for HIF-1α expression. β-Actin was used as the internal control.

Table S3. Primer sequences used for RT-PCR

NANOG-F	5′-CAAAGGCAAACAACCCACTT-3′
NANOG-R	5′-TCTGCTGGAGGCTGAGGTAT-3′
OCT4-F	5′-GTATTCAGCCAAACGACCATC-3′
OCT4-R	5′-CTGGTTCGCTTTCTCTTTCG-3′
MYC-1201F	5′-GGCAAAAGGTCAGAGTCTGG-3′
MYC-1409R	5′-GTGCATTTTCGGTTGTTGC-3′
CCND1-519F	5′-GAACAAACAGATCATCCGCAAAC-3′
CCND1-684R	5′-GCGGTAGTAGGACAGGAAGTTG-3′
CDH2-890F	5′-CAGATAGCCCGGTTTCATTTGA-3′
CDH2-1053R	5′-CAGGCTTTGATCCCTCAGGAA-3′
EPCAM-127F	5′-AATCGTCAATGCCAGTGTACTT-3′
EPCAM-304R	5′-TCTCATCGCAGTCAGGATCATAA-3′

Supplementary Methods

Western Blot Analysis

Protein was extracted from cells using ice-cold RIPA buffer (50 mM Tris HCl (pH 7.4), 150 mM NaCl, 1 mM EDTA, 1% (v/v) Triton-X 100, 0.1% (w/v) SDS) supplemented with phenylmethylsulfonyl fluoride (PMSF) and protease inhibitor cocktail (2 mM AEBSF, 1 mM EDTA, 130 μM bestatin, 14 μM E-64, 1 μM leupeptin, 0.3 μM aprotinin). Protein concentrations were determined using a bicinchoninic acid (BCA) assay according to the manufacturer's instructions. Equivalent amounts of

protein were separated on 12% SDS-PAGE gels and transferred to polyvinylidene difluoride (PVDF) membranes (Millipore, Billerica, MA, USA). Membranes were blocked at room temperature with 5% nonfat milk in Tris-buffered saline (TBS) containing 0.1% Tween 20 (TBS-T) for 1 hour. Primary antibodies were diluted 1:2000 and incubated overnight at 4°C. Secondary antibodies diluted 1:5000 were added and incubated at room temperature for 1 h. Signals were detected using ECL detection reagent (Millipore) following the manufacturer's instructions. The primary antibodies used were as follows: mouse anti-β-catenin, mouse anti-lamin A/C (BD Biosciences, CA, USA), mouse anti-c-myc, mouse anti-cyclin D1, rabbit anti-HDAC1, rabbit anti-HIF-1α and rabbit anti-β-actin (GeneTex, CA, USA). Horseradish peroxidase-conjugated rabbit anti-mouse or goat anti-rabbit secondary antibodies (GeneTex, CA, USA) were used as appropriate.

TCF/LEF Luciferase Assay

The pGL4.21 [luc2P/Puro] vector was purchased from Promega (Madison, WI, USA). The M50 Super 8× TOPflash and M51 Super 8× FOPFlash vectors were obtained from the Randall T. Moon laboratory. The sequences of the TCF/LEF binding sites in the M50 Super 8× TOPflash and M51 Super 8× FOPFlash vectors were cloned into the pGL4.21 vector. The pGL4.21-TOPflash and pGL4.21-FOPflash vectors were generated and used in this study. CL1-0, H1437, and CL1-0/CDDP cells were transfected with the pGL4.21-TOPflash vector and incubated for 2 days. Then, cells were treated with the indicated concentration of emetine for 6 hours. Firefly and Renilla luciferase activities were measured with a Dual-Glo Luciferase Assay System (Promega). The relative activity of TOPflash or FOPflash was normalized to Renilla luciferase activity and indicated the fold change in Wnt/β-catenin pathway activation.

Immunocytochemical (ICC) Staining

CL1-0 and CL1-0/CDDP cells were seeded on 12-mm glass coverslips and cultured overnight. Cells were washed with phosphate-buffered saline (PBS) and fixed in 4% formaldehyde solution (Sigma-Aldrich) at room temperature for 20 min. Fixed cells were washed and permeabilized with 0.5% Triton X-100 in PBS for 15 min at room temperature. Permeabilized cells were washed and blocked with 1% BSA in PBS overnight at 4 °C. The primary antibody against mouse anti-β-catenin (BD Biosciences, CA, USA) was added at a 1:200 dilution and incubated overnight at 4 °C. Cells probed with the primary antibody were washed with PBS three times. Secondary antibodies were diluted 1:300 and incubated at room temperature for 1 h.

Cells were washed and stained with Hoechst 33342 (bisbenzimide H33342 trihydrochloride) (Sigma-Aldrich) to visualize nuclei. Stained cells on coverslips were mounted to slides using Fluoromount Aqueous Mounting Medium (Sigma-Aldrich). Fluorescence images were acquired with an LSM 880 confocal laser scanning microscope with Airyscan (Carl Zeiss Inc., Germany). FITC-conjugated anti-mouse IgG (Bethyl Laboratories, TX, USA) and DyLight 594-labeled anti-rabbit IgG (GeneTex) secondary antibodies were used as appropriate.

Nuclear Extraction

Nuclear extracts were prepared using a Nuclear Extraction Kit 2900 (Millipore Corporation, Billerica, MA, USA) according to the manufacturer's instructions. After treatment with distilled water or emetine, cells were washed with PBS and collected. Cytoplasmic proteins were isolated in cytoplasmic lysis buffer containing 0.5 mM DTT and protease inhibitor. Cell pellets were washed again with cytoplasmic lysis buffer. Nuclear proteins were isolated in nuclear lysis buffer containing 0.5 mM DTT and a protease inhibitor. The concentration of nuclear protein was measured with a Pierce BCA Protein Assay Kit (Thermo, Waltham, MA, USA).
